# Acute posterior multifocal placoid pigment epitheliopathy (APMPPE)

**DOI:** 10.1186/s12348-021-00263-1

**Published:** 2021-09-16

**Authors:** Ilaria Testi, Sandra Vermeirsch, Carlos Pavesio

**Affiliations:** Department of Uveitis, Moorfields Eye Hospital, National Health Service Foundation Trust, 162 City Rd, Old Street, London, EC1V 2PD UK

**Keywords:** Acute posterior multifocal placoid pigment epitheliopathy, APMPPE, Placoid epiteliopathy, Imaging, Optical coherence tomography angiography

## Abstract

**Background:**

Acute posterior multifocal placoid pigment epitheliopathy (APMPPE) is a rare inflammatory eye disease, affecting the inner choroid and the outer retina. Recent advances in multimodal imaging have been important in the understanding of the pathophysiology of the disease, allowing a better characterization of the morphology of this condition.

**Methods:**

Narrative review.

**Results:**

In this review, a comprehensive overview of clinical features, imaging findings, treatment management, and long-term outcomes of patients with APMPPE will be provided.

**Conclusions:**

Although APMPPE was originally believed to be a self-limited condition with a good prognosis, the disease can be recurrent and result in significant loss of vision function. Fundus imaging plays an important role in the diagnosis and management of the disease, allowing to evaluate response to treatment and onset of complications.

## Introduction

Acute posterior multifocal placoid pigment epitheliopathy (APMPPE) is a rare inflammatory disease, classified as part of the spectrum of the white dot syndromes, a terminology presently revised. Today it is classified within the group of choriocapillaritis diseases. The disorder was first described by Gass in 1968 as multiple, discrete, cream-coloured lesions located in the posterior pole of three young women complaining of painless visual reduction. At that time, the retinal pigment epithelium (RPE) was considered to be the primary site of inflammation [1]. However, as in the case of posterior uveitis, multimodal imaging played a key role in the characterisation of disease phenotype, and advances in imaging technologies have resulted in a better understanding of its pathophysiology. Multimodal imaging features in APMPPE support the most likely primary inflammatory involvement of the choriocapillaris with secondary photoreceptor disruption, resulting in the characteristic distinctive clinical phenotype of the disease.

This review article provides a comprehensive overview of the clinical features, imaging findings, treatment management, and long-term outcomes of the lesions that characterize patients with APMPPE.

## Epidemiology and demographics

APMPPE is a rare disease, occurring predominantly in young healthy adults. The disorder is found equally between men and women, with a male to female ratio of 1.2:1 [2]. Data combined from published case reports identified 27 years as the mean age of onset of the disorder, with a range between the first and seventh decade, and over three quarters of cases occurring between 16 and 40 years of age [2, 3]. The disease is more common in Caucasian people [2, 3].

## Etiopathogenesis

The pathophysiology of the disease is supported by the characteristic findings on multimodal imaging, showing that APMPPE primarily affects the choriocapillaris and inner choroid, resulting in secondary changes to the outer retina and RPE [4–7]. However, the exact underlying etiologic mechanism is unknown, although an immune driven nature has been hypothesized based on the following associations. The disease is often preceded by a viral prodrome, therefore the hypothesis of its association with a presumed infective illness is suggestive [2, 8–12]. In addition, numerous cases of APMPPE have been reported in patients with a positive tuberculosis immunological test, suggesting an indirect mechanism eliciting an intraocular immune response [2, 3, 13]. The disease has also been linked with pre-existing autoimmune and autoinflammatory diseases, including psoriasis, sarcoidosis, erythema nodosum, eczema, and diabetes mellitus, and cases of patients developing such conditions after being diagnosed with APMPPE have also been described [14–16]. An increased incidence of both class I HLA antigen (HLA-B7) and class II HLA antigen (HLA-DR2) with a relative higher risk of 3.38 and 3.32 of developing ocular disease, respectively, has been reported in APMPPE patients [17]. The immune driven nature is also supported by the development of the disease after immunization with vaccines, including hepatitis B, varicella, polio, and tetanus, suggesting a possible interplay between vaccines and specific T-cell receptors of the host immune system [18–23].

## Clinical manifestations

**Fig. 1:**
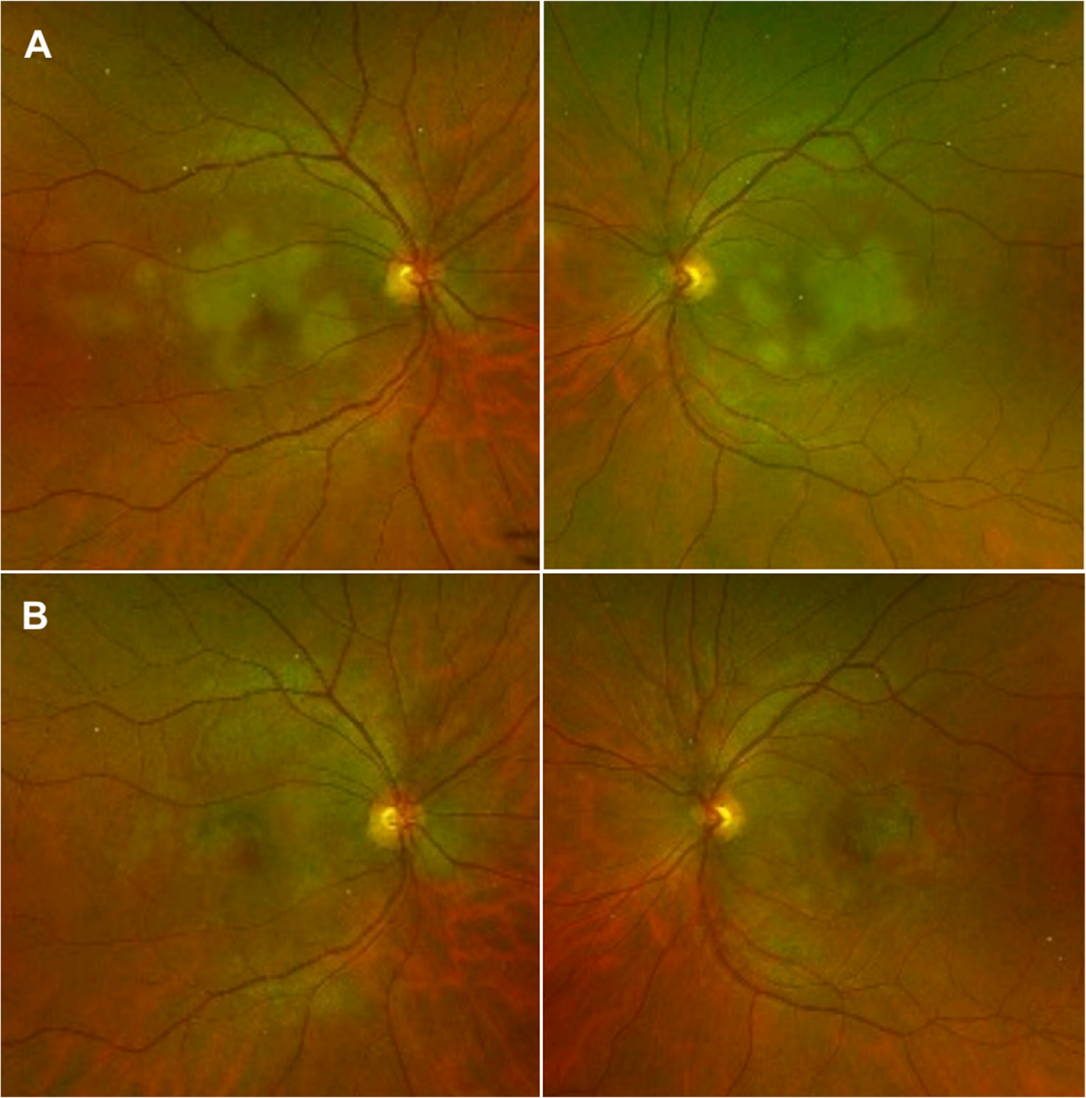
Pseudocolour fundus photograph of acute posterior multifocal placoid pigment epitheliopathy (APMPPE). (**A**) Acute stage - Bilateral multifocal, yellowish creamy, placoid lesions, located at the posterior pole. (**B**) Inactive stage – Bilateral resolution of placoid lesions resulted in hyperpigmentation and chorioretinal atrophy

APMPPE clinically manifests with multifocal, yellowish creamy, subretinal, placoid lesions, located posteriorly to the equator, generally within the posterior pole (Fig. 1**A**). Patients complain of bilateral sudden and painless visual loss, photopsias and paracentral scotomas. In most cases patients have bilateral disease with the second eye affected within a few days or weeks following the fellow eye. The lesions tend to gradually fade over few weeks, resulting in hyperpigmentation and chorioretinal atrophy (Fig. 1**B**). New lesions may appear up to three weeks following the onset, coexisting with older lesions. The disease may be associated with minimal inflammatory reaction in the vitreous cavity, usually in the absence of anterior uveitis. Papillitis and retinal vasculitis may be present [2, 3, 24–26].

Although APMPPE is a primary ocular disease, neurological and systemic manifestations can also occur. Central nervous system complications include cerebral vasculitis, cavernous sinus thrombosis, meningoencephalitis, aseptic meningitis, stroke or transient ischaemic attack, seizures, sixth-cranial-nerve palsy, transient hearing loss and headaches [10, 27–30]. In addition, peripheral neuropathy has recently been described [27]. In a retrospective review of 56 cases diagnosed with APMPPE, Algahtani et al. reported cerebral vasculitis as the most common neurological complication, affecting 50% of the patients, following by isolate headaches in 26.8% [27].

## Ancillary imaging

### Fundus photography

Fundus photography is useful to document the appearance of APMPPE lesions and determine their morphological evolution and associated changes, including atrophy and hyperpigmentation (Fig. 1). In addition, onset of new lesions can be detected over the course of the disease. Thus, serial fundus photography from acute onset to the stage of healing is a useful tool that allows an objective assessment of disease activity, and can also help in detecting potential complications, including development of choroidal neovascularization clinically manifesting as haemorrhage contiguous to the scar [31–35].

### Fundus fluorescein angiography and Indocyanine green angiography

Active APMPPE lesions show hypofluorescence in the early phases of fundus fluorescein angiography (FFA), but will become hyperfluorescent in the late frames. The early hypofluorescence is likely to represent poor perfusion or delayed filling of the choriocapillaris rather than choroidal signal attenuation from the overlying outer retina and/or RPE thickening/oedema, as initially believed by Gass [1, 5, 24–26]. The late hyperfluorescence is the result of the change in the polarity of the RPE, affected by ischaemia, resulting in a deficit of pumping that interferes with the movement of the fluid from the retina to the choroid. Another hypothesis has been put forward indicating that profound ischaemia of the outer retina could produce reactionary hyperpermeability and exudation from retinal vessels [36, 37]. Healed lesions may demonstrate hyper and hypofluorescence throughout the exam, reflecting RPE disturbance, namely window defect due to RPE atrophy and pigmentary changes.

On indocyanine green angiography (ICGA) active placoid lesions appear as hypofluorescent spots across all phases of the exam, likely due to poor perfusion of the choriocapillaris [5, 6, 38, 39]. The ICGA signal usually normalizes as APMPPE lesions resolve. Although ICGA and FFA findings have been interpreted as suggesti of a choroidal origin to the disease, the alternative hypothesis suggesting a RPE origin with subsequent potential ICGA and early FFA signal attenuation by swollen/thickened RPE could potentially contribute to this presentation [39].

### Optical coherence tomography

**Fig. 2:**
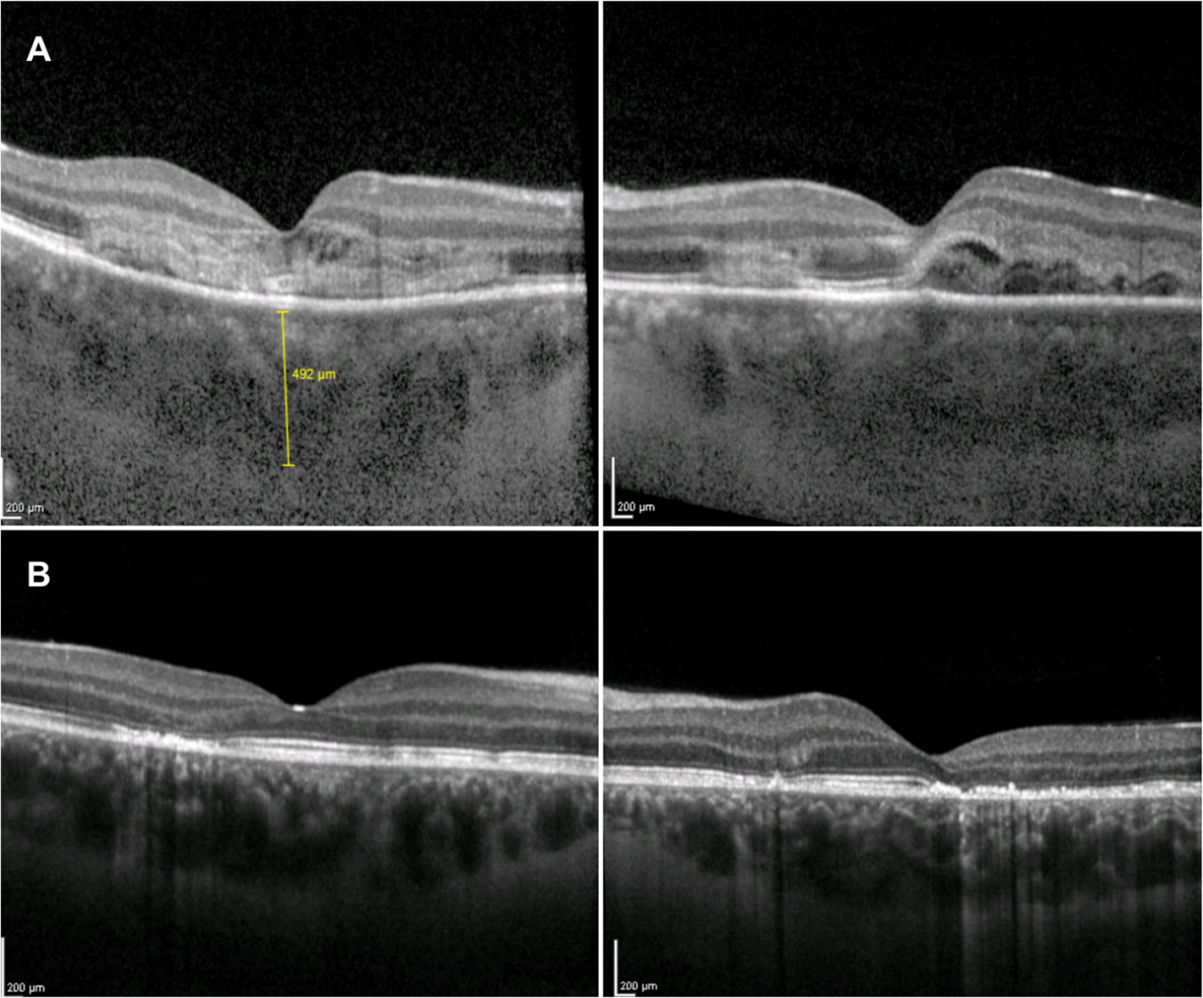
Enhanced depth imaging optical coherence tomography (EDI-OCT) of acute posterior multifocal placoid pigment epitheliopathy (APMPPE). (**A**) Active stage - Bilateral disruption of the outer retinal and ellipsoid zone, with hyperreflectivity of outer retinal layers and retinal pigment epithelium (RPE), and bilateral increase in choroidal thickness. (**B**) Inactive stage - Resolution of the placoid lesions with disappearance of the hyperreflectivity of the outer retina, resulting in either partial restoration of its appearance or focal areas of photoreceptors/RPE atrophy; bilateral normalization of choroidal thickness

On spectral-domain optical coherence tomography (SD-OCT), the placoid lesions in the acute stage appear as disruption of the outer retinal and ellipsoid zone, with the presence of hyperreflective material at the level of the outer retinal layers and RPE (Fig. 2**A**) [24–26, 40]. Over the course of resolution of the placoid lesions, the hyperreflectivity of the outer retina disappears, resulting in either partial restoration of its appearance or focal areas of photoreceptors/RPE atrophy (Fig. 2**B**) [41, 42].

Goldenberg described four distinct OCT phases of APMPPE, beginning with a dome-shaped elevation with disruption of the photoreceptor junction, that flattens soon after [43]. Two weeks later, the second stage is characterized by the detection of a distinct separation between the photoreceptor junction and the RPE. The third stage, occurring six weeks after disease onset, is characterized by increased RPE hyperreflectivity and union of the RPE and photoreceptor junction, followed by the resolution phase, starting at three months post disease onset, with the reformation of two distinct visible layers of photoreceptors and RPE. However, focal areas of photoreceptors/RPE atrophy can develop.

Accumulation of associated subretinal fluid might occur in APMPPE, and the presence of exudative retinal detachment mimicking acute Vogt-Koyanagi-Harada has been reported [44, 45]. Recently, Kohli et al. described three cases of APMPPE characterized in the acute phase by a SD-OCT revealing a splitting of the ellipsoid zone suggestive of bacillary layer detachment [46]. All cases showed complete resolution within one week. It has been hypothesized that the underlying impairment of choroidal vascularization could result into the photoreceptor (bacillary layer) stress and splitting. Since the photoreceptors get their nutrients from the choriocapillaris, the inner choroidal ischemia characterizing the acute stage of the disease could lead to the splitting of the bacillary layer. Once the inflammation resolves and the choriocapillaris perfusion improves, the bacillary detachment then regresses [47–50]. Another possible explanation for the bacillary detachment could be the change in the polarity of the RPE, affected by ischaemia, resulting in a deficit of pumping that interferes with the movement of the fluid from the retina to the choroid.

During the acute phase of the disease enhanced depth imaging optical coherence tomography (EDI-OCT) shows choroidal thickening, improving with the resolution of the placoid lesions (Fig. 2) [44–46].

### Optical coherence tomography angiography

Recent advances in the field of ocular imaging and development of optical coherence tomography angiography (OCT-A) have allowed a better assessment of APMPPE morphology. OCT-A visualizes retinal and choroidal circulations, and, providing segmented en-face images of blood flow in the retinal capillary plexuses and choriocapillaris, has allowed a better understanding of the pathophysiology of the choroidal vascular involvement characterizing the disease**.**

In patients with APMPPE, OCT-A demonstrates areas of decreased flow/flow void at the level of the choriocapillaris [39, 51–53]. These areas of flow deficit correlate closely with the ischemic lesions manifesting as early hypofluorescent on FFA and hypofluorescent throughout the exam on ICGA [39, 51–53]. In addition, corresponding areas of outer retinal disruption on SD-OCT co-localize with the areas of reduction of choriocapillaris flow on OCT-A. However, such areas of outer retinal changes on SD-OCT co-localize with greater areas of choriocapillaris flow deficit on OCT-A, supporting the hypothesis that APMPPE is primarily caused by an ischemic event occurring at the level of the inner choroid and secondary affecting the outer retina and RPE with photoreceptor disruption [39, 53]. OCT-A demonstrates a reversible inner choroidal hypoperfusion, showing normalisation of choroidal vasculature during follow-up.

### Fundus autofluorescence

Fundus autofluorescence (FAF) acts a measure of RPE function, and autofluorescence characteristics have been recognized as a marker of disease activity, allowing prediction of therapy response and visual prognosis [54].

**Fig. 3:**
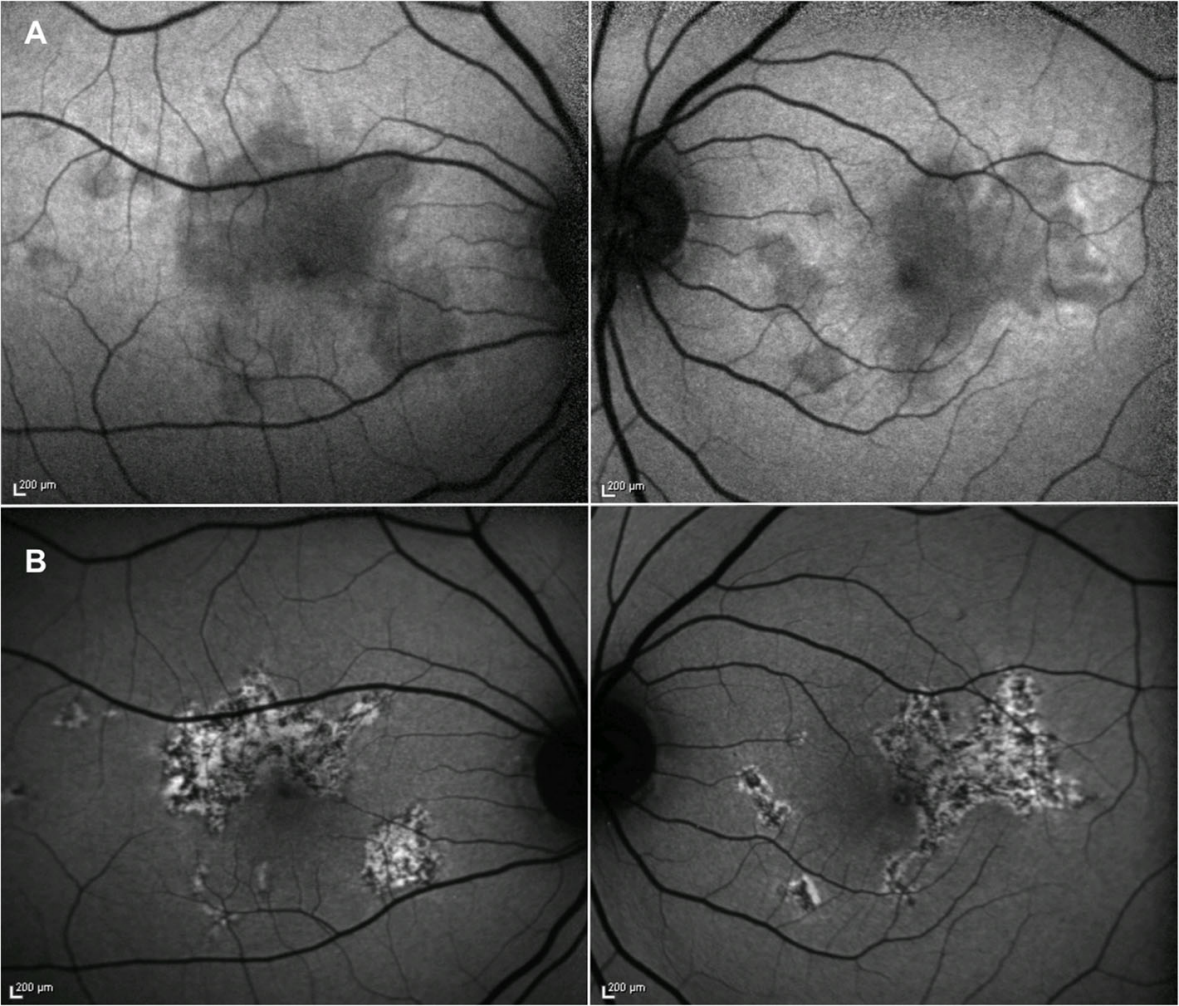
Fundus autofluorescence of acute posterior multifocal placoid pigment epitheliopathy (APMPPE). (**A**) Active stage - Bilateral initial hypoautofluorescence corresponding to the hyper-reflectance of the outer retinal layers on optical coherence tomography (OCT). (**B**) Inactive stage - Progressive increase in hyperautofluorescence, resulting in a mixed pattern of hypoautofluorescence and hyperautofluorescence

APMPPE lesions show initial hypoautofluorescence corresponding to the hyper-reflectance of the outer retinal layers on SD-OCT, likely secondary to its masking effect (Fig. 3**A**) [55]. A progressive increase in hyperautofluorescence is observed later on in the course of the disease when outer retinal findings progress to disruption of the RPE layer, with a mixed pattern of hypoautofluorescence and hyperautofluorescence (Fig. 3**B**) [55, 56]. Later in the disease course, a more homogenous hypoautofluorescent pattern suggesting focal areas of photoreceptors/RPE atrophy might develop [55, 56].

Imaging features of APMPPE are described in Table 1**.**

**Table 1:** Imaging features of acute posterior multifocal placoid pigment epitheliopathy (APMPPE)

		FFA	ICGA	FAF	SD-OCT	EDI-OCT	OCT-A
APMPPE	Active disease	Early hypofluorescence, late hyperfluorescence	Early and late hypofluorescence	Initial hypoautofluorescence, with progressive increase in hyperautofluorescence	Disruption of the outer retinal and ellipsoid zone, with hyperreflective material at the level of the outer retinal layers and RPE	Choroidal thickening	Areas of flow void at the level of the choriocapillaris
	Healed disease	Early and late hyper and hypofluorescence reflecting RPE disturbance (window defect and pigmentary changes)	Normalisation	Mixed pattern of hypoautofluorescence and hyperautofluorescence, followed by homogenous hypoautofluorescent	Either partial restoration of outer retina or focal areas of photoreceptors/RPE atrophy	Normalisation of choroidal thickness	Normalisation of choriocapillaris flow

FFA: fundus fluorescein angiography; ICGA: indocyanine angiography; FAF: fundus autofluorescence; SD-OCT: spectral domain optical coherence tomography; EDI-OCT: enhanced deep imaging optical coherence tomography; OCT-A: optical coherence tomography angiography; RPE: retinal pigment epithelium

## Laboratory and radiologic investigations

Although the diagnosis of APMPPE is based on clinical features and multimodal imaging findings, an infectious aetiology and associated systemic causes need to be ruled out. Laboratory investigations must include angiotensin converting enzyme (ACE) level and tuberculin intradermal reaction/Quantiferon test, considering the potential association of the disease with sarcoid or tuberculosis [2, 3, 13, 14, 16]. Considering the wide range of manifestations of ocular syphilis, exclusion of this condition should be part of the investigations.

In case of significant headaches and on suspicion of neurologic involvement, patients must be investigated with brain magnetic resonance imaging (MRI) and cerebrospinal fluid (CSF) examination. If cerebral vasculitis is suspected, MR angiography (MRA) is required [28].

## Management and prognosis

In the acute stage of the disease, the use of anti-inflammatory medications, including systemic steroids, is recommended to reduce the retinal damage. Generally, patients are started on a dose of 1 mg/kg/day of oral Prednisolone, which is gradually tapered over several weeks, pending on clinical response supported by imaging findings. In recurrent disease, the introduction of a second-line steroid-sparing agent, such as antimetabolite, usually mycophenolate mofetil, may be necessary. If an underlying infectious aetiology is identified, specific antimicrobic therapy is required, usually alongside anti-inflammatory therapy. Patients with focal neurological symptoms require intravenous methylprednisolone, followed by a tapering course of oral steroids for at least 3 months. Patients with APMPPE and isolated headaches with a CSF pleocytosis should be treated with oral steroids.

Although the original description categorized APMPPE as a self-limited condition with a good prognosis, the reality is that the disease can be recurrent and result in significant visual loss, which can be the result of both direct central macular damage and the presence of large scotomas in a paracentral location. In a retrospective analysis and review of literature conducted by Fiore et al. in patients with APMPPE, at last follow-up visit visual acuity was 20/25 or less in 42.3% eyes and 20/40 or less in 23.7% eyes [57]. However, 71.9% eyes were symptomatic at last follow-up visit, underlining that patients with APMPPE might experience incomplete visual recovery [57].

## Method of literature search

Literature search was undertaken in June 2021. A search of Medline, using PubMed and Google Scholar, was performed with no date restriction. The search used the following keywords: ‘acute posterior multifocal placoid pigment epitheliopathy’, ‘APMPPE’, ‘placoid epiteliopathy’, ‘white dot syndromes’. Articles were selected based on clinical importance and categorized according to their relevance to the following section headings: epidemiology, demographics, etiopathogenesis, clinical features, imaging, management, and prognosis. Articles in languages other than English were considered if they provided an English abstract which could be used for screening their relevance.

## Data Availability

Not applicable.
